# Supplementary material for: Consumer Awareness, Attitudes and Preferences Towards Heritage Cereals
Source: Foods. 2020 Jun 4;9(6):742. doi: 10.3390/foods9060742 (PMC7353474; doi:10.3390/foods9060742)
Supplement: Supplementary file 1 [file foods-09-00742-s001.pdf]

Supplementary File S1: Consumer Questionnaire

Q1. Age?
_____years

Q2. Gender
A. man
B. woman
C. different identity

Q3. Academic institute
A. Kristianstad University
B. Swedish Agricultural University

Q4. Which cereal products do you consume at least once a week? You may choose more than one alternative:
A. Pasta
B. Bread
C. Porridge
D. Rice
E. Cooked cereals
F. Breakfast-cereals/muesli
G. Cakes/cookies
H. None
I. Others (please specify) ------------------

Q5. Which of the following heritage cereals are you aware of?
A. Emmers
B. Oland wheat
C. Wheat
D. Svedjerag
E. Einkorn
F. Dinkle/Spelt
G. Kamut
H. Hallands wheat,
I. None, others (please specify) ------------------

Q6. Do you consume bread?
A. Yes
B. No

Q7. Which type of bread do you eat?

Please specify ------------------

Q8. How often do you bake bread at home? Please specify in the scale 1 to 4 where 1 = always and 4= never.

1 ----- 2 ----- 3 ----- 4

Appendix 1

Q9. How often do you buy bread in a bakery? Please specify in the scale 1 to 4 where 1 = always and 4= never.

1 ----- 2 ----- 3 ----- 4

Q10. How often do you buy bread in a grocery store? Please specify in the scale 1 to 4 where 1 = always and 4= never.

1 ----- 2 ----- 3 ----- 4

Q11. Rate the importance of each of the following factors with regards to bread quality. Scale 1-5, where 1= not-important, 5=very important

A. Appearance
B. Taste/Flavour
C. Texture
D. Odour/Aroma
E. Based on sourdough
F. Based on wholemeal
G. Health aspects
H. Origin of cereal grain
I. Cereal grain being organic
J. Price
K. Brand
L. Freshness
M. Shelf-life

Q12. Would you consider buying bread or other cereal-products based on heritage cereals?

A. Yes
B. No

Q13. Which heritage cereal products would you consider buying in the future? You may choose more than one alternative:

A. Pasta
B. Bread
C. Porridge
D. Cooking cereals
E. Breakfast cereals
F. Cookies
G. flour
H. None of the above
I. Others (please specify) -----------------

Q14. Would you be willing to pay more for products based on heritage cereals?

A. Yes
B. No
